# Concepts and methods for transcriptome-wide prediction of chemical messenger RNA modifications with machine learning

**DOI:** 10.1093/bib/bbad163

**Published:** 2023-05-03

**Authors:** Pablo Acera Mateos, You Zhou, Kathi Zarnack, Eduardo Eyras

**Affiliations:** EMBL Australia Partner Laboratory Network at the Australian National University, Canberra, Australia; The Shine-Dalgarno Centre for RNA Innovation, The John Curtin School of Medical Research, Australian National University, Canberra, Australia; The Centre for Computational Biomedical Sciences, The John Curtin School of Medical Research, Australian National University, Canberra, Australia; Buchmann Institute for Molecular Life Sciences (BMLS), Goethe University Frankfurt, Max-von-Laue-Str. 15, 60438 Frankfurt a.M., Germany; Institute of Molecular Biosciences, Goethe University Frankfurt, Max-von-Laue-Str. 15, 60438 Frankfurt a.M., Germany; Buchmann Institute for Molecular Life Sciences (BMLS), Goethe University Frankfurt, Max-von-Laue-Str. 15, 60438 Frankfurt a.M., Germany; Institute of Molecular Biosciences, Goethe University Frankfurt, Max-von-Laue-Str. 15, 60438 Frankfurt a.M., Germany; EMBL Australia Partner Laboratory Network at the Australian National University, Canberra, Australia; The Shine-Dalgarno Centre for RNA Innovation, The John Curtin School of Medical Research, Australian National University, Canberra, Australia; The Centre for Computational Biomedical Sciences, The John Curtin School of Medical Research, Australian National University, Canberra, Australia

**Keywords:** machine learning, deep learning, RNA modifications, epitranscriptomics, direct RNA sequencing, miCLIP

## Abstract

The expanding field of epitranscriptomics might rival the epigenome in the diversity of biological processes impacted. In recent years, the development of new high-throughput experimental and computational techniques has been a key driving force in discovering the properties of RNA modifications. Machine learning applications, such as for classification, clustering or *de novo* identification, have been critical in these advances. Nonetheless, various challenges remain before the full potential of machine learning for epitranscriptomics can be leveraged. In this review, we provide a comprehensive survey of machine learning methods to detect RNA modifications using diverse input data sources. We describe strategies to train and test machine learning methods and to encode and interpret features that are relevant for epitranscriptomics. Finally, we identify some of the current challenges and open questions about RNA modification analysis, including the ambiguity in predicting RNA modifications in transcript isoforms or in single nucleotides, or the lack of complete ground truth sets to test RNA modifications. We believe this review will inspire and benefit the rapidly developing field of epitranscriptomics in addressing the current limitations through the effective use of machine learning.

## INTRODUCTION

The first evidence of an internal chemical modification in RNA was found more than 60 years ago when pseudouridine was discovered as a fifth nucleotide in yeast RNA [1]. The development and cheapening of high-throughput sequencing technologies have accelerated our capacity to study these modifications in a transcriptome-wide manner. Today, more than 150 internal RNA modifications have been discovered that can decorate RNA molecules [2]. Some of them have been observed transcriptome-wide in protein-coding RNAs (mRNAs), such as N6-methyladenosine (m^6^A) [3, 4], 5-methylcytosine (m^5^C) [5], 5-hydroxymethylcytosine (hm^5^C) [6], pseudouridine (Ψ) [7–9] and inosine (I) [10], with different deposition frequencies across the transcriptome [11]. Some modifications were found to be reversible [12], suggesting a role in dynamically regulating processes of RNA metabolism, such as splicing, translation, export and stability [13–16]. One of the most abundant and well-characterized modifications in eukaryotic mRNA is m6A. The deposition of m6A mainly occurs within DRACH sequence motifs (D = A, G or T; R = A or G; H = A, C or T) and shows a strong enrichment around stop codons [3, 4]. The presence of m6A is dynamically regulated by the METTL3-METTL14 methyltransferase ‘writer’ complex that deposits m6A on mRNA, as well as ‘reader’ proteins that bind m6A and ‘eraser’ enzymes that remove m6A [17]. Through writers, readers and erasers, m6A is involved in the regulation of possibly all steps of RNA processing and function, thereby impacting major physiological functions such as cell differentiation and development [18, 19]. Even though our understanding of the roles and localization of m6A and other modifications is improving, there are still major gaps in the knowledge of the epitranscriptome. One of the bottlenecks is the lack of rapid, reliable and universal methods for detecting these modifications transcriptome-wide.

A critical advance in epitranscriptomics has been enabled by the development of transcriptome-wide experimental methods to detect modifications at single-nucleotide and/or single-molecule resolution in a quantitative manner. These range from a targeted detection of RNA modifications by specific antibodies or enzymes to directly reading RNA modifications in RNA molecules using direct RNA sequencing (DRS). Computational tools have been essential in processing and analyzing the experimental outputs to identify RNA modifications [20]. Particularly, machine learning (ML) has been effective at harnessing these large and complex data. This has been facilitated by the availability of ML software libraries that are easy to use and incorporate graphics processing unit (GPU)-accelerated algorithmic implementations [21].

ML has increased our ability to perform complex prediction tasks that are difficult to manually or formally define because of the large number of parameters or the many special cases or exceptions [22]. ML algorithms can process experimental input data and automatically identify the right features to tackle problems such as classification, regression or clustering. Moreover, these algorithms are extremely flexible and adaptable. Properties of the ML methods such as inductive biases, network architectures and loss functions can be effectively combined with biological knowledge, such as RNA sequence motifs, mRNA transcript features and RNA secondary structures to study the complexity of the epitranscriptome. A plethora of ML algorithms has been developed in the last 15 years to predict RNA modifications and discover their biological functions. Recent findings include the role of m6A in the splicing efficiency of adenovirus RNA [23], the coordinated deposition of m6A in m5C modifications in human cell and mouse brain transcripts [24], and the surprising invariance of pseudouridine modifications in rRNA under stress conditions and across translational repertoires [25].

**Figure 1:**
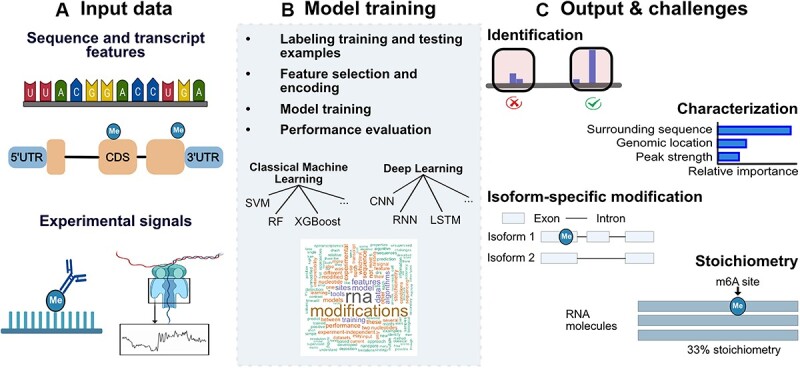
Transcriptome-wide prediction of chemical messenger RNA modifications with ML. The identification of chemical modifications in messenger RNA (mRNA) involves (**A**) reading sequence information alone or in combination with experimental data, (**B**) training and testing of ML methods and (**C**) analysis of the predicted outputs in terms of properties such as the localization of the RNA modifications, association with specific mRNA isoforms, stoichiometry and functional characterization. XGBoost, eXtreme Gradient Boosting; LSTM, long short-term memory. The figure was created with BioRender.com.

Previous reviews describing ML approaches for RNA modification detection have focused either on technology-specific methods, like those based on long-read sequencing [26, 27], or have solely described methods that are based on sequence classification [20, 22, 28, 29]. In this review, we show the progression of technologies, data types and algorithms to predict RNA modifications, separating them into two major strategies, experiment-independent, which refers to methods that identify modifications using only the reference sequence, and experiment-based, which refers to methods that process high-throughput experimental data in addition to the reference sequence to identify the modifications (Figure 1A). We focus on the different steps and modeling decisions to create efficient algorithms for RNA modification detection and highlight best practices (Figure 1B). Finally, we examine the current technology-specific and ML-related challenges in the field of epitranscriptomics and possible routes to overcome these (Figure 1C).

## MODELING BIOLOGY: CLASSICAL MACHINE LEARNING AND DEEP LEARNING

In this review, we make the distinction between two types of ML algorithms: deep learning (DL), which employs neural networks (NNs) with multiple layers, and classical ML, which includes methods such as linear models, random forests (RFs) and support vector machines (SVMs). One of the main differences between the two types is feature extraction, i.e. the identification and transformation of the input data for the ML task. In classical ML, feature extraction is usually performed separately prior to using the algorithm. In contrast, DL algorithms can perform ‘end-to-end learning’, creating an internal representation of the input data in their latent space and performing feature extraction automatically. One of the advantages of DL methods is that they can be specifically adapted to suit initial assumptions about the nature of the input data distribution, also called inductive bias. For instance, convolutional neural networks (CNNs) and recurrent neural networks (RNNs) incorporate properties that make them especially suitable for modeling data with a strong spatial component, such as images or sequential data. Similarly, geometric DL methods can take inputs in the form of a graph, which makes them suitable for RNA secondary structure or gene co-expression modeling [30–32]. Although DL methods can outperform classical ML methods in conditions in which sufficient training data are available, DL algorithms tend to overfit due to their many parameters and capacity to fit the data, declining their performance outside their training space. Moreover, GPUs are often required to meet DL’s higher computing requirements. In contrast, classical ML algorithms are easier and faster to prototype and implement, and provide broader user accessibility and better interpretability. Overall, the choice of the ML algorithm depends on various factors, such as the nature of the data, the complexity of the task, the available resources and the expertise of the users. Each algorithm has its strengths and weaknesses and choosing the right one can significantly impact a tool’s performance.

## CONCEPTS IN FOCUS

Multiple relevant biological and computational elements must be considered for the identification of RNA modifications with ML methods. From the epitranscriptomics perspective, algorithms should ideally be capable of identifying RNA modifications at single-nucleotide resolution, associating RNA modifications with transcript isoforms, and accurately estimating their stoichiometry, i.e. the fraction of copies of a given RNA molecule that harbor an RNA modification at a given site. On the other hand, from the ML perspective, algorithmic expertise must be combined with domain knowledge to guide training, testing, feature extraction, feature selection and interpretability. We will discuss these topics in the next sections for the two major strategies for detecting RNA modifications with ML, which we refer to as experiment-independent and experiment-based methods. Experiment-based methods use data from high-throughput experiments during ML inference that are either specifically designed to identify or enrich a specific modification (reviewed in [33]), or that can be queried to detect modifications, such as nanopore long-read sequencing data [34]. In contrast, experiment-independent methods do not necessitate such experimental data and are able to operate using the reference sequence alone. In experiment-independent methods, annotated RNA sequences and transcript characteristics are often used as features to predict RNA modifications. Once the ML model has been trained on already-known, experimentally determined RNA modification sites, the predictions are made solely based on the RNA sequences and transcript features. As this makes it possible to learn about RNA modifications without an additional experiment, these tools are essential to meet the demand for information on RNA modifications in many studies. However, experiment-independent methods are ‘blind’ to any sample changes or perturbations and will predict the same RNA modification sites independently of cell type, phenotype or condition. This limitation can be overcome by the rapidly growing field of experiment-based methods. Here, features acquired from a laboratory experiment—either targeted to a specific RNA modification or covering all modifications—are incorporated as model input. This approach can hence be used to detect RNA modifications from specific conditions, species or phenotypes, potentially uncovering modifications unique to a particular condition.

## EXPERIMENT-INDEPENDENT ML METHODS TO PREDICT RNA MODIFICATIONS

Experiment-independent ML methods for the prediction of RNA modifications are based on the observation that many RNA modifications show a certain sequence and positional preference in their deposition, as exemplified by the preferred occurrence of m6A modifications within DRACH motifs and close to stop codons [3, 4, 35] or away from splice sites and splice site-like motifs [36, 37]. This suggests that RNA sequence alone may determine to a certain extent the presence of RNA modifications and can hence be exploited to predict their deposition in a transcript without the need to perform an experiment. Experiment-independent approaches generally take experimentally determined RNA modification sites as a starting point to train ML algorithms, taking as input the RNA sequence and possibly additional features. Many ML algorithms have been implemented in experiment-independent tools (Table 1). SVM is the most common algorithm used in the tools surveyed, followed by the decision tree-based algorithms RF and XGBoost. The more recently developed tools use DL, including CNNs as the most widely used, as well as Bidirectional Gated Recurrent Units, and RNNs. We describe below different strategies for training and testing these tools, including the selection and encoding of features, and introduce the performance metrics used in experiment-independent algorithms. Furthermore, we describe interpretability techniques to investigate the major determinants of RNA modifications. Due to the similarity of approaches to build experiment-independent tools for predicting different RNA modifications, in this review we focus on tools for detecting m6A RNA modifications as an example. Other experiment-independent tools for RNA modification prediction, such as iRNA-m7G [38], have been surveyed before [22].

**Table 1:** Survey of tools using experiment-independent ML models

Tool	Balance of testing set	Input(s)	Encoding scheme	Algorithm	Model interpretation	Performance metrics	MCC	Ref.
AthMethPre	Unbalanced	Nucleotide sequence	One-hot, *k*-mer	SVM	NA	ROC, PRC, MCC	0.39	[84]
BERMP	Unbalanced	Nucleotide sequence	ENAC, RNA word embedding	DL	NA	ROC, MCC	0.31–0.72	[43]
DeepPromise	Unbalanced	Nucleotide sequence	ENAC, one-hot, RNA word embedding	DL	Motif extraction from kernels	ROC, MCC	0.48, 0.57	[29]
TDm6A	Unbalanced	Nucleotide sequence	One-hot	DL	Motif extraction from kernels	ROC, ACC, MCC	0.30–0.43	[44]
iM6A	NA	Nucleotide sequence	One-hot	DL	Single nucleotide saturation mutagenesis	ROC, PRC	NA	[36]
DeepM6ASeq	Balanced	Nucleotide sequence	One-hot	DL	Motif extraction from kernels, saliency map	ROC, PRC, ACC, MCC, F1 score	0.53	[49]
RAM-ESVM	Balanced	Nucleotide sequence	PseDNC, motif features	SVM	NA	ACC, MCC	0.57	[51]
Gene2vec	Unbalanced	Nucleotide sequence	One-hot, neighboring methylation state, RNA word embedding, Gene2vec	DL	Motif extraction from kernels	ROC, PRC, MCC	0.45–0.50	[45]
Tool	Balance of testing set	Input(s)	Encoding scheme	Algorithm	Model interpretation	Performance metrics	MCC	Ref.
Deep-m6A	Unbalanced	Nucleotide sequence	One-hot	DL	NA	ROC, PRC	NA	[85]
m6Apred	Unbalanced	Nucleotide sequence	NCP, ANF	SVM	NA	ROC, ACC	NA	[41]
M6ATH	Balanced	Nucleotide sequence	NCP, ANF	SVM	NA	ROC, PRC, ACC, MCC	0.72	[86]
RAM-NPPS	Balanced in *Saccharomyces cerevisiae* and *Arabidopsis thaliana*, unbalanced in human	Nucleotide sequence	RFE, FSDI, MRMD	SVM	NA	ROC, PRC, ACC, MCC	0.59–0.79	[87]
HMpre	Unbalanced	Nucleotide sequence, SNP, relative position, entropy	One-hot, CPD, *k*-mer	XGBoost	Feature importance	Precision, recall, MCC, F1 score	0.33	[56]
M6A-HPCS	Balanced	Nucleotide sequence	PCPM, PseDNC, AC, CC	SVM	Relative gain	ROC, ACC, MCC	0.45	[50]
DeepM6APred	Balanced	Nucleotide sequence	One-hot, deep features, NPPS	SVM	NA	ACC, MCC	0.61	[47]
iRNA(m6A)-PseDNC	Balanced	Nucleotide sequence	PseDNC	SVM	NA	ROC, ACC, MCC	0.83	[52]
iRNA-Methyl	Balanced	Nucleotide sequence	PseDNC, RNA property parameters	SVM	NA	ACC, MCC	0.29	[40]
M6AMRFS	Balanced	Nucleotide sequence	Dinucleotide binary, local position-specific dinucleotide frequency	XGBoost	NA	ACC, MCC	0.49–0.83	[46]
M6APred-EL	Balanced	Nucleotide sequence	PS(k-mer)NP, PCPs, RFHC-GACs	SVM	NA	ROC, PRC, ACC, MCC	0.62	[88]
MethyRNA	Balanced	Nucleotide sequence	NCP, ANF	SVM	NA	ACC	NA	[48]
Tool	Balance of testing set	Input(s)	Encoding scheme	Algorithm	Model interpretation	Performance metrics	MCC	Ref.
pRNAm-PC	Balanced	Nucleotide sequence	PCPM	SVM	NA	ROC, ACC, MCC	0.40	[89]
RFAthM6A	Balanced	Nucleotide sequence	PSNSP, PSDSP, KSNPF, *k*-mer	RF	Feature importance	ROC, MCC	0.71–0.73	[55]
RNA-MethylPred	Balanced	Nucleotide sequence	BPB, DNC, KNN score	SVM	NA	ROC, ACC, MCC	0.45	[54]
RNAMethPre	Unbalanced	Nucleotide sequence, relative position, MFE	One-hot, *k*-mer, MFE	SVM	NA	ROC, PRC, MCC	0.30–0.50	[57]
SRAMP	Unbalanced	Nucleotide sequence, secondary structure	One-hot, KNN score, spectrum	RF	Feature importance	ROC, PRC, MCC	0.21–0.41	[42]
TargetM6A	Balanced	Nucleotide sequence	PSNP, PSDP, *k*-mer	SVM	NA	ROC, ACC, MCC	0.21	[90]
WHISTLE	Unbalanced	Nucleotide sequence, 35 genomic features	NCP, ANF, genome-derived features	RF	Feature importance	ROC	NA	[39]
MultiRM	Balanced	Nucleotide sequence	One-hot, word2vec, Hidden Markov Model	DL	Integrated gradient, attention weights	ROC, PRC, ACC, MCC	0.31–0.85	[60]
iRNA-m7G	Balanced	Nucleotide sequence	PseDNC, SSC, NPF	SVM	NA	ROC, ACC, MCC	0.8	[38]

*Note:* ROC, receiver operating characteristic curve; PRC, precision-recall curve; XGBoost, eXtreme Gradient Boosting; ENAC, enhanced nucleic acid content; PseDNC, pseudo dinucleotide composition; NCP, nucleotide chemical property; ANF, accumulated nucleotide frequency; RFE, recursive feature elimination; FSDI, feature selection based on discernibility and independence of a feature; MRMD, maximal relevance and maximal distance; CPD, chemical property with density; PCPM, physicochemical property matrix; AC, auto-covariance; CC, cross-covariance; NPPS, nucleotide pair position specificity; PSNP, position-specific nucleotide sequence profile; PCPs, physical–chemical properties; RFHC-GACs, ring-function-hydrogen-chemical properties without GAC; PSNSP, position-specific nucleotide sequence profile; PSDSP, position-specific dinucleotide sequence profile; KSNPF, K-spaced nucleotide pair frequencies; BPB, bi-profile bayes; DNC, dinucleotide composition; MFE, minimum free energy; PSDP, position-specific dinucleotide propensity; SSC, secondary structure component; NPF, nucleotide property and frequency. DeepPromise can detect m1A and m6A, MultiRM is designed to detect 12 types of RNA modification, iRNA-m7G can detect m7G and all the remaining tools in Table 1 are designed to detect m6A. For tools that were tested on multiple sample types (e.g. BERMP in four different species, TDm6A in four different cell lines), we provide the range of MCC values obtained.

### Labeling training and testing examples

**Figure 2:**
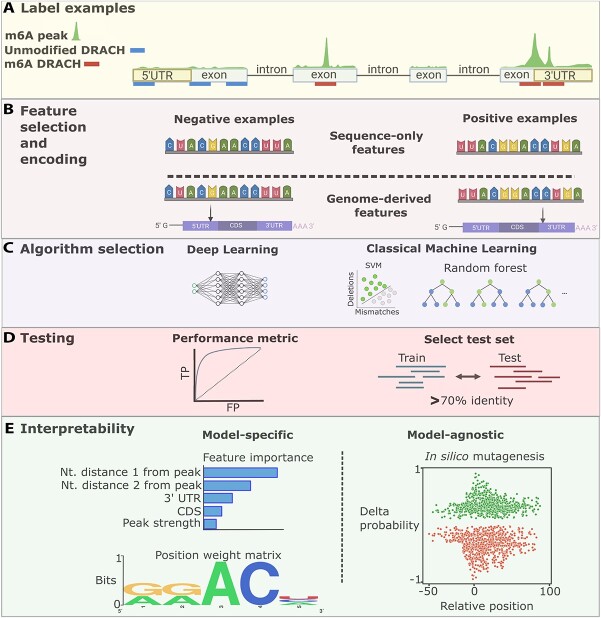
Experiment-independent ML approaches to predict RNA modifications. The schematic illustrates a generic approach using the example of m6A. (**A**) RNA sequences are labeled according to whether they contain the RNA modification based on existing experimental data. (**B**) Experiment-independent ML methods can be built on sequence-only features, which are based on nucleotide strings from the RNA sequence, or on more general genome-derived features, which contain a mix of RNA sequence and other features, such as RNA secondary structure, relative location within the transcript or evolutionary conservation of the modified position. (**C**) Both DL and classical ML methods, such as RFs and SVMs have been used in experiment-independent RNA modification detection. (**D**) Choosing the right testing data and accuracy metrics is essential for the correct estimation of algorithm performance. Left: The AUROC is a commonly used performance metric based on true positives (TP) and false positives (FP). Right: A common approach for separating training and testing sequences maintains at least 70% of identity between the two groups. (**E**) Interpretability methods can be either model-specific or model-agnostic. The left panel shows two examples of model-specific interpretability: the relative importance of several features, and a sequence logo (position weight matrix) obtained using the activation values from the first layer of a CNN model for m6A detection. The right panel introduces *in silico* saturation mutagenesis as an example of a model-agnostic method for interpretability. The figure was created with BioRender.com.

Correct labeling of the training and testing examples is an essential—albeit not trivial—step for RNA modification modeling. Most experiment-independent tools surveyed in this review take the union of detected modification sites from several experiments as positive examples for training and testing (Figure 2A). The exception is WHISTLE [39], which defines as positive examples those sites that have been identified in at least two datasets. This is expected to increase the robustness of the positive examples in the training and testing data.

In contrast to the positive examples, the strategies for collecting negative examples, i.e. sequences not containing the RNA modification, are more heterogeneous across tools. The most common strategy for m6A prediction extracts DRACH/GAC/RRAC/A sites either from the complete transcriptome or only from those transcripts that harbor the positive examples [39–47] (Figure 2A). In contrast, methods like MethyRNA [48] and DeepM6ASeq [49] select the negative examples from the flanking sequence of the positive examples regardless of whether these contain a DRACH motif. Both approaches generally identify an excess of negative examples, leading to an imbalanced setup for training and testing (Table 1). This could result in algorithms with poor predictive accuracy for the minority class or in an overestimated performance, as discussed below.

### Feature selection and encoding

Experiment-independent ML models follow two main approaches regarding the selection of features: they either use only features based on the nucleotide sequence, referred to as sequence-only features, or incorporate other genomic features, referred to as genome-derived features (Figure 2B). The majority of tools use sequence-only features but rely on different feature encoding strategies, i.e. how the input data are presented to the ML model. The most implemented encoding is simply the nucleotide sequences surrounding modified and unmodified sites. Other tools also include *k*-mer frequencies (with *k* generally varying between 1 and 4 nucleotides) in the surrounding sequences, RNA secondary structure-related features or even physicochemical properties of the nucleotides. For example, M6A-HPCS [50], RAM-ESVM [51], iRNA-Methyl [40] and iRNA(m6A)-PseDNC [52] implement a pseudo K-tuple nucleotide composition [53], which encodes the sequence and physicochemical properties of K-tuple nucleotides to capture both local and global sequence patterns. Other tools like RNA-MethylPred [54] and SRAMP [42] compare the input sequences with known positive and negative examples using the *k*-nearest neighbor (kNN) algorithm. The proportion of positive samples in the kNNs is then used as the kNN similarity score to make the prediction. Although some tools compare the performance among certain encoding schemes [29, 43, 46, 51, 55], there is no systematic benchmarking to date that comprehensively compares all encoding schemes.

Methods based on genome-derived features (Figure 2B) use positional information related to the modified nucleotide and the transcript structure, such as distance to exon-intron boundaries or the stop codon. The incorporation of such features is generally motivated by the observation that modifications like m6A have a strong positional bias. Methods like WHISTLE [39], HMpre [56] and RNAMethPre [57] use the relative location of sites on the transcript as a feature for training their models. Among these, WHISTLE incorporates the largest number of genome-derived features, including the relative location within the transcript, the length of the transcript region, the distance to exon-intron boundaries, the evolutionary conservation of the candidate site and its flanking region and properties of the genes or transcripts containing the modified sites, such as being miRNA target genes or housekeeping genes. Other tools like HMpre [56] use single nucleotide polymorphisms (SNPs) within the sequence as a feature, under the assumption that functionally relevant modification sites should be depleted of SNPs.

### Data preparation and performance metrics for experiment-independent tools

In most ML scenarios (Figure 2C), the exact data distribution on which the algorithm will be used is unknown; hence, a testing set is used to approximate the future performance of the algorithm. Creating a robust and independent testing set is essential to properly estimate the algorithm’s future performance. In experiment-independent RNA modification prediction, different strategies have been used to create an independent testing set. Randomly splitting into training and testing data or leave-one-out strategies may be suboptimal as they do not allow controlling the similarity between training and testing data. Different approaches may help to identify overfitting and give a less biased estimation of ML performance (Figure 2D). Tools like DeepM6ASeq [49], TDm6A [44] and SRAMP [42] use sequence identity to remove highly redundant sequences between training and testing data. Another training–testing split design implemented by WHISTLE [39] uses cross-validation based on m6A sites from different experiments as a held-out dataset. Finally, an alternative strategy to obtain an independent testing set, which is commonly used in genomics, but not yet for RNA modification detection, is to leave out data from one chromosome as the testing set [58].

ML performance metrics are essential to assess how the predictions of a given algorithm resemble the ground truth when tested on an independent testing set prepared as described above. There is no single universal performance metric, as different metrics can describe different advantages and limitations of an algorithm. Metrics like the area under the receiver operating characteristic curve (AUROC) and overall accuracy (ACC) are popular to measure the model’s performance. However, the training and testing data in experiment-independent methods are often unbalanced, as they tend to contain more negative than positive examples, which can lead to an underestimation of the false positive rate (FPR). The AUROC uses the FPR, which may not be realistic on an unbalanced testing set. Therefore, choosing the appropriate metric is essential to avoid over- or underestimation of the performance of a model. The area under a precision-recall curve (AUPRC) measures the ability to predict positive examples considering the false positives and thereby represents a more realistic measure of an algorithm’s performance. Another summary metric that is robust to unbalanced datasets is the Matthews correlation coefficient (MCC), which generates a high score only if the prediction obtained good results in all four categories of the confusion matrix (true positives, false negatives, true negatives and false positives). In our survey, AUROC, ACC and MCC are the most commonly used performance metrics by experiment-independent predictors (Table 1). Despite being a more robust metric, AUPRC is only provided by a subset of the surveyed tools.

### Interpretability

Interpretability refers to the degree to which we can understand the decisions made by an ML method and gain biological insights from it (Figure 2E). Classical ML algorithms are generally easier to interpret as they directly operate on previously defined and selected features. For instance, SVMs and tree-based methods define the predictions in terms of conditions over features directly identifiable in the input data, which can be more easily interpreted using well-established approaches [59]. Because of this, classical ML algorithms may be desirable in scenarios in which interpretability is the main objective. In our survey, 11 out of the 29 surveyed tools implement interpretability strategies (Table 1). Two approaches are generally used, which can be discriminated into model-specific and model-agnostic.

#### Model-specific interpretability methods

Model-specific interpretability methods take advantage of certain properties of the ML algorithm to interrogate its predictions. One of the most common techniques for epitranscriptomics algorithms is feature importance from decision tree-based algorithms such as RF and XGBoost (Figure 2E). The feature importance scores provide a rapid and easy way to understand the global relative impact of each feature for the classification task. The algorithms to calculate the feature importance scores are usually built into the ML packages.

NNs trained to classify modified and unmodified RNA sequences can also be interrogated for interpretability. CNNs use kernels, i.e. a fixed-size weight matrix, to process the input from one layer and extract important features for classification. In the first CNN layer, kernels scan through all the positions of the input vector and calculate an activation value for each position. Activation values increase with the relevance of a combination of input features on the classification score. Tools like TDm6A [44], Gene2vec [45] and DeepM6ASeq [49] extract nucleotides from the input with the highest activation values from positive examples, which are then used to generate a position weight matrix to visualize important sequence motifs for classification (Figure 2E). These can be used, for example, to compare with known binding motifs for RNA-binding proteins which may be relevant for the deposition of the RNA modification.

DL models with attention have also been used to identify RNA sequence motifs important for model classification. MultiRM [60] is the first DL model to simultaneously detect putative sites for 12 RNA modifications (m6A, m1A, m5C, m5U, m6Am, m7G, Ψ, I, Am, Cm, Gm and Um) from input sequences. The algorithm is based on a long short-term memory network that maps the input RNA sequences to 12 context vectors, each corresponding to the output prediction for one RNA modification. MultiRM uses attention weights and integrated gradients to explain visually how the model makes specific decisions. As with CNNs, this method evaluates each input’s contribution to the prediction and assigns higher scores to important nucleotides in the input sequences.

#### Model-agnostic interpretability methods

Model-agnostic interpretability methods can be used with any ML model, as their applicability does not depend on the specific properties of the ML algorithm. For instance, to understand the relative contribution of each feature for identifying m6A modifications, M6A-HPCS [50] uses an empirical method called relative gains. For calculating the relative gain, the model is first trained with all the features and a performance metric is estimated, such as accuracy. Then, one feature is removed at a time for the training and testing steps. The difference in the performance metric between the full and the reduced model is defined as the relative gain for the removed feature.

As an alternative strategy, iM6A [36] implements *in silico* saturation mutagenesis to understand how individual nucleotides influence the ML-based predictions of m6A (Figure 2E). First, high-confidence predicted m6A sites are extracted based on their model-predicted posterior probability and their relative location within the transcripts. Then, each flanking nucleotide is substituted with the other three possible nucleotides to calculate the difference in the predicted probability of the model before and after the ‘mutation’. Using this approach, the initial study found that the mRNA features determining m6A deposition preferentially reside within 50 nucleotides downstream of the m^6^A sites.

## EXPERIMENT-BASED ML METHODS TO DETECT RNA MODIFICATIONS

While experiment-independent approaches are valuable for learning about potential RNA modifications, they are inherently static and cannot account for dynamic RNA modifications that specifically occur in a cell type, phenotype or condition. Experimental data are therefore essential to close this gap and provide a real-time picture of RNA modifications. An increasing number of experimental high-throughput techniques, mostly based on high-throughput sequencing technologies, allow for the transcriptome-wide identification of RNA modifications in a sample-specific manner [33]. Building on these innovations, experiment-based ML models have been developed for the detection of RNA modifications either from targeted experiments or from DRS.

### m6A detection from targeted experiments

**Figure 3:**
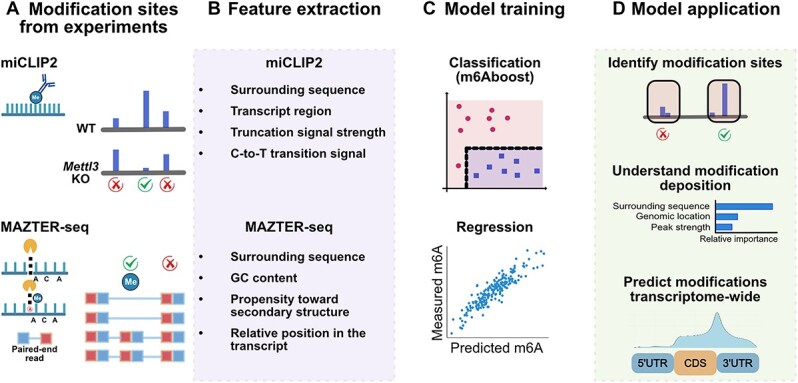
The workflow of ML methods for data from targeted experiments. (**A**) m6Aboost [61] is trained on miCLIP2 data. Differential methylation analysis upon *Mettl3* KO is used to identify positive and negative examples. The experimental protocol MAZTER-seq [63] identifies the m6A sites using the methylation-sensitive RNase MazF which cleaves at ACA motifs only if these are not methylated. (**B**) Multiple RNA sequence and additional features are extracted from the m6A sites identified by miCLIP2 and MAZTER-seq. (**C**) Classical ML models are trained on the extracted features. (**D**) The model can be used to identify RNA modification sites in a transcriptome-wide manner from new experimental data. Interpretability techniques enable investigating the determinants of m6A deposition. The figure was created with BioRender.com.

To date, two approaches have been presented that use classical ML methods to leverage information from targeted experiments for m6A (Figure 3). The first tool, m6Aboost [61], was developed to extract reliable m6A sites from experimental data obtained by m6A individual-nucleotide resolution UV crosslinking and immunoprecipitation 2 (miCLIP2). miCLIP2 [61], like the original protocol miCLIP [62], employs an m6A-specific antibody combined with a targeted library preparation and sequencing strategy to detect m6A sites at single-nucleotide resolution. However, due to limited antibody selectivity, the experimental data contain excessive background signals at non-m6A sites. Simply removing the background by filtering for DRACH motifs—a commonly employed strategy—may overlook non-DRACH m6A sites. To overcome this limitation, m6Aboost employs a decision tree-based AdaBoost model to predict m6A sites from miCLIP2 data based on RNA sequence, genomic context and experimental features. The ML model is trained on positive and negative examples identified from a differential methylation analysis upon *Mettl3* knockout (KO). m6Aboost is thereby able to detect both non-DRACH and DRACH m6A sites with very high performance on an independent testing set.

Classical ML has also been employed to investigate m6A sites detected by the antibody-independent experimental method MAZTER-seq [63]. MAZTER-seq is based on the ability of the RNase MazF to cleave RNA at unmethylated anti-centromere antibody (ACA) motifs but not at their methylated counterparts. The m6A stoichiometry is then quantified from the number of sequencing reads that begin, terminate and read through each transcriptomic ACA site. After identifying m6A sites from MAZTER-seq data, the RNA secondary structure, relative position, surrounding sequence and guanine and cytosine (GC) content of these m6A sites are used to build a linear model to predict m6A deposition. This approach found that nearly 50% of the variability in m6A levels can be primarily explained by the local sequence context, with minor contributions from RNA secondary structure and the proximity of the site to the 3′ end of the gene. The ML model can also be used to predict m6A sites in the transcriptome *de novo*.

### Detection of RNA modifications from DRS

**Figure 4:**
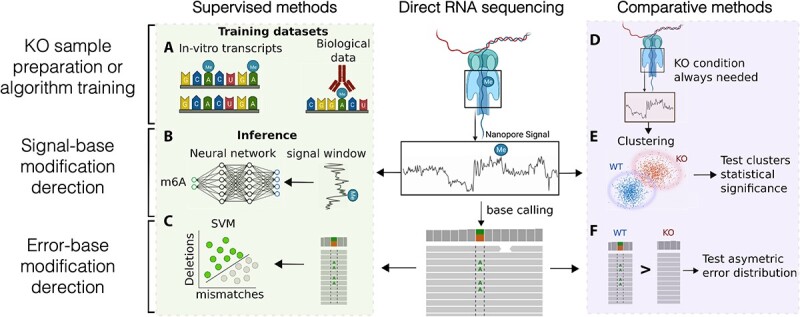
ML methods for RNA modification detection using DRS. (**A**) IVTs and experimentally determined modification sites from cell lines (symbolized by an antibody) can be used as training data for supervised learning methods to detect RNA modifications in nanopore DRS data. (**B**) Supervised learning methods use signal properties to detect RNA modifications. (**C**) SVMs can be used to detect RNA modifications by modeling basecalling errors. (**D**) Most unsupervised learning methods detecting RNA modifications need a background sample, usually a condition with a KO of the modifying enzyme. (**E**) Unsupervised clustering of signal properties such as mean signal value per 5-mer or dwell time can be used to detect RNA modifications. For unsupervised clustering, WT and KO (in the image) or background conditions are used to group signals into modified and unmodified clusters. (**F**) Statistical tests can also be used to test the asymmetric distributions of errors between normal and background conditions to detect RNA modifications. The figure was created with BioRender.com.

Nanopore DRS is an emerging technology that allows the sequencing of native RNA molecules at a transcriptomic scale [34]. In DRS, RNA molecules are translocated through a nanopore with the help of a motor protein. While the RNA molecules are passing through the pore, the alterations of an ionic current are measured, which approximately correspond to a combination of five nucleotides in the current technology. Importantly, modified RNA nucleotides can alter the ionic current and the time through the pore (dwell time) differently from unmodified nucleotides [34]. DRS thus opens the possibility of detecting chemical modifications directly in the transcripts but also brings up new experimental and computational challenges. Even though DRS has only been available for a relatively short period of time (since 2018), there are already multiple tools for RNA modification detection (Table 2, Figure 4). In the following, we describe these tools in terms of their approach to detecting RNA modifications and discuss their advantages and limitations.

**Table 2:** Tools to detect RNA modification from nanopore DRS data

Tool	Algorithm	Features	Predicts on individual reads	Needs two conditions (WT versus KO)	Predicts stoichiometry	Predicts on motif	RNA modifications	Ref.
CHEUI	CNN	Signal values	Yes	No	Yes	Any	m6A/m5C	[24]
EpiNano (comparative mode)	SVM	Errors	No	Yes	No	Any	Diff	[64]
EpiNano (SVM)	SVM	Errors	No	No	No	Any	m6A	[64]
Tombo (comparison mode)	Statistical test	Signals	No	Yes	No	Any	Diff	[67]
Tombo (alternative mode)	Alternative model	Signals	No	No	No	Any	m5C	[67]
ELIGOS	Fisher’s exact test	Errors	No	Yes	No	Any	Diff	[65]
Nanocompore	GMM	Dwelling time and signal values	No	Yes	Yes	Any	Diff	[68]
xPore	GMM	Signal values	No	Yes	Yes	Any	Diff	[69]
MINES	RF	Features from Tombo ‘de novo’ detection	No	No	No	DRACH	m6A	[73]
JACUSA2	Beta-binomial distribution	Nucleotide base substitution, deletion and insertion scores	No	Yes	No	Any	Diff	[66]
DRUMMER	G-test	Basecalling error rates at both exon, and isoform level	No	Yes	No	Any	Diff	[23]
Tool	Algorithm	Features	Predicts on individual reads	Needs two conditions (WT versus KO)	Predicts stoichiometry	Predicts on motif	RNA modifications	Ref.
Nanom6A	XGBoost		No	No	Yes	RRACH	m6A	[74]
NanoRMS	kNN supervised models	Signal intensity, basecall probabilities and dwell time	No	No	Yes	Any	Ψ	[25]
EpiNano-RMS (comparative mode)	kNN or K-means	Sts signal intensity, basecall probability and dwell time	No	Yes	Yes		Depends on other methods	[64]
Yanocomp	Multivariate GMMs	Signal values	No	Yes	Yes	Any	Diff	[70]
Penguin	SVM	Reference 5-mer, and mean value, standard deviation, and length of 5-mer signals	Yes	No	Yes	Any	Ψ	[91]
nanoDoc	CNN	Current level, and dwell time	Yes	Yes	Yes	Any	Diff	[71]
m6Anet	CNN	Mean nanopore signal, standard deviation and dwell time	Yes	No	Yes	DRACH	m6A	[75]
Dinopore	CNN	Mean, standard deviation, dwell time, base qualities, mismatches and deletions	No	No	Yes	Any	A-I	[77]
DENA	RNN	Mean, median, standard deviation, dwell time and base quality	Yes	No	Yes	RRACH	m6A	[76]

#### Comparative approaches to detect differential RNA modifications

Several algorithms detect RNA modification by comparing features of the nanopore sequencing reads between two conditions—most often in an unsupervised manner—where one of them is expected to contain lower levels of one or more RNA modifications. Many such methods use error patterns to predict modifications. The underlying idea is that RNA basecalling algorithms, which predict the RNA sequence from the nanopore sequencing signal, have not been trained with this modification information. It is thus expected that they will present a low prediction probability and a higher error rate on and around modified nucleotides. Based on this principle, samples with and without modifications are expected to have a non-symmetric set of errors that can be leveraged to detect RNA modifications. This strategy is used by EpiNano (comparative mode) [64], ELIGOS [65], DRUMMER [23] and JACUSA2 [66], which compare the basecalling error profile between DRS from the sample of interest and a control sample depleted of modifications, e.g. *in vitro* transcripts (IVTs) or RNA from a sample after a KO or knockdown (KD) of an RNA-modifying enzyme. ELIGOS [65] also uses other control samples such as cDNA reads, which do not harbor RNA modifications due to the reverse transcription step to generate cDNA, or an RNA background error model, empirically calculated from errors using IVTs. While this gives ELIGOS the potential advantage of not needing to sequence a second sample to find RNA modifications, the accuracy of these alternative controls has not been comprehensively tested. Despite the effectiveness of using basecalling errors to identify modified bases, it has been seen that using only error frequencies is a suboptimal strategy to predict stoichiometry [25]. Moreover, these methods will be dependent on the specific basecalling method used.

Error profiles are not the only features that can be leveraged in a comparison between two conditions. Other features such as average signal intensity values and dwell time can be used to compare a sample and a reference to detect modifications. Non-error features improve the detection of RNA modifications and correlate better with stoichiometry than error-based features [25, 64]. For instance, Tombo [67] compares the mean signal values from two samples at each reference position using the module *level_sample_compare.* Nanocompore [68] clusters mean signal values and dwell time from two conditions, one of them a control with lower levels of an RNA modification, using a two-component Gaussian mixture model (GMM) followed by a logistic regression test. This provides an improvement over using a single statistical test to determine if one of the samples was modified, reducing the number of false positives [68]. xPore [69] also implements two Gaussian distributions to cluster two samples in an unsupervised way, while also introducing prior information regarding the theoretical signal distribution of unmodified RNAs. These priors help to guide the model estimation of Gaussian parameters and show better performance than EpiNano [64] and Tombo [67]. xPore [69] also shows improved stoichiometry prediction compared to previous methods albeit at the cost of a high FPR [24]. Yanocomp [70] uses a similar methodology but introduces a 5-nt sliding window to fit multivariate GMMs, taking advantage of the observation that each RNA modification may affect the nanopore signal differently at multiple nucleotide positions. It also adds an additional component to control for possible outliers. However, no comparisons with other tools have been made so far. Finally, nanoRMS [25] is the only comparative tool that combines signal values, dwell time and basecalling error profiles between modified and unmodified samples to detect modifications.

In contrast to the previously described tools, nanoDoc [71] uses a supervised DL approach to detect RNA modifications. It implements two parallel CNNs that share the same weights. Each CNN takes nanopore signal and dwell time values corresponding to a 5-mer and transforms each of the two inputs into a 16-dimensional vector. Then, the Euclidean distance is measured between the two output vectors to infer how different the original inputs are. With this strategy, nanoDoc processes pairs of complex multidimensional input signals and infers a distance function between them by transforming the inputs into a pair of vectors that can be easily compared, e.g. through Euclidean distance, to determine if the input corresponds to a modified nucleotide.

One of the advantages of the comparative and unsupervised approaches is that, in principle, they can be used to detect the modification status of RNA without requiring a ground truth. Their main limitation is that, since these approaches require a control or KO/KD sample, the prediction is inherently indirect. Moreover, the necessity for KO/KD samples increases the complexity and costs of the experiment and may not always be feasible. An additional problem is that the removal of an RNA modification may generate secondary effects like the depletion of other RNA modifications. For instance, it has been shown that the depletion of NSUN2 to remove m5C leads to a reduction of hm5C [65]. Furthermore, modifications may depend on multiple enzymes, as exemplified by m5C which can be deposited by multiple enzymes [72]. In this situation, inactivation of only one of them may result in a partial detection of the m5C sites in the transcriptome. This makes it challenging to fully characterize the epitranscriptome using the comparative approach.

#### Supervised learning to detect RNA modifications in one condition

Supervised learning algorithms can be trained to recognize patterns linked with modified and unmodified nucleotides in future unseen samples. In contrast to the comparative methods described above, supervised methods directly predict the specific modification, rather than inferring it from a comparison between two conditions. Several tools have been developed using this approach. Besides the comparative mode described above, EpiNano [64] can also predict m6A from the base quality scores, mismatches and deletion frequencies of nucleotides in one sample using an SVM. The SVM was trained using modified (m6A) and unmodified IVTs and tested using both IVTs and RNA from yeast cells with and without *IME4*, a yeast gene encoding a METTL3 homolog.

In a different approach to predict m6A sites, MINES [73] uses an RF on the output of Tombo’s *de novo* mode to classify DRACH motifs into modified or unmodified [67]. MINES was trained using DRS data combined with miCLIP-identified m6A sites from HEK293 and HeLa cells. One limitation of MINES is that it does not predict the modification status of individual DRS reads or stoichiometry. Similarly, Nanom6A [74] implements an XGBoost model trained using IVTs with and without m6A to classify RRACH motifs in the annotation reference into modified or unmodified. Nanom6A was the first algorithm using nanopore and supervised learning that provided information about the stoichiometry of the predicted site.

Several other methods apply DL to model DRS data. For instance, m6Anet [75] is a feedforward NN model (multiple layer perceptron) that uses previously identified transcriptomic m6A sites and Multiple Instance Learning for training. In this approach, the model is trained to classify m6A-modified and unmodified sites without explicitly providing information about individual reads, but rather using groups of reads associated with previously selected modified or unmodified sites. While m6Anet shows higher accuracy than Nanom6A [74] and EpiNano [64] using an *in vivo* HEK293 dataset, it only identifies m6A sites in DRACH motifs and has a low precision. Another method that uses previously identified m6A sites to train an NN is DENA [76]. DENA’s training is based on comparing basecalling errors in individual reads from wild type (WT) and m6A methyltransferase KO conditions in *Arabidopsis thaliana* at various RRACH motifs. The mean and median values of sites with significant error differences are used as predictive features. Other similar DL methods have been developed to detect A-to-I editing sites from nanopore DRS data [77].

In parallel, CHEUI was developed as a two-stage prediction model that detects both m6A and m5C from the DRS signals [24]. In the first stage, a CNN identifies m6A or m5C at single nucleotide positions in individual reads using the signals from the 9-mer surrounding the nucleotide. In the second stage, CHEUI uses a second CNN to process all the individual read predictions from the first model at a given transcriptomic site to predict the stoichiometry and modification probability of that site. CHEUI was trained using IVTs and tested using independent IVTs and several *in vivo* samples. It was shown to outperform xPore [69], Nanocompore [68], Tombo [67] and NanoRMS [25] in stoichiometry prediction accuracy, the number of true positives detected and the control of the false positives. Another advantage of CHEUI is that it can predict m6A or m5C in any sequence context. This advantage made it possible to confirm the presence of DRACH-independent m6A sites and the sequence and structural features of m5C sites that are dependent or independent of NSUN2, one of the m5C-modifying enzymes.

ML models have also been developed for other RNA modifications, such as pseudouridine (Ψ) [78]. Using a small number of synthetic RNAs with and without pseudouridine, sequence-specific models such as RF or XGBoost were trained using 35 features, including the basecalling quality scores, and deletions of the bases on and around the candidate site. These models showed high accuracy and stoichiometry correlation but limited generalization to sequences outside those used for training.

## CURRENT CHALLENGES

### Isoform-specific RNA modifications

Identifying RNA modifications at isoform-specific resolution is one of the major open challenges in epitranscriptomics. Experimental methods based on short-read sequencing have the inherent limitation that most reads cannot be confidently assigned to a specific isoform. As a consequence, when RNA modifications are predicted in transcript regions that occur in multiple isoforms, it is challenging to identify whether all isoforms, or only a subset of them, are modified. Long reads from nanopore DRS can span the full length of a transcript isoform, circumventing the ambiguity of short reads. In principle, experimental-based methods based on DRS data could take this advantage to assign modifications to specific transcript isoforms [24]. In practice, however, there are still technical limitations in DRS, such as a considerable number of truncated reads resulting in a coverage decay toward the 5′ end [79]. Current and new experimental and computational methods will play a crucial role in overcoming these hurdles and obtaining a better understanding of isoform-specific RNA modifications.

### Stoichiometry

To quantitatively characterize and fully understand the functional roles of RNA modifications, it will be crucial not only to accurately predict their positions but also their stoichiometry, i.e. the fraction of copies of a given RNA molecule that harbor an RNA modification at a given site. Current evidence suggests that stoichiometry can be variable across sites and modification types in the same condition or even in the same transcript [24]. Moreover, for m6A, there is evidence that at least for a subset of sites, the stoichiometry is ‘hard-coded’ in the RNA sequence context [63]. As outlined above, several ML tools already offer the possibility to estimate stoichiometry, for instance, via read-level predictions of RNA modifications on DRS data. However, reliable experimental data to train and benchmark these approaches remain a major bottleneck to date. Recently, new promising experimental methods have been developed based on nucleotide conversion that accomplish accurate transcriptome-wide estimates of the m6A stoichiometry [80, 81]. The resulting data show a high correlation with DRS-based estimates [24] and promise excellent potential for investigating stoichiometry in the future.

### Cell type and condition-specific RNA modifications

An important question in epitranscriptomics is whether RNA modifications vary across cell types, tissues and other conditions and whether these differences are related to regulatory mechanisms or phenotypic states. As experiment-independent methods do not incorporate condition-specific information, they will detect the same RNA modifications in different cell types and conditions, hence providing a ‘static’ description of the epitranscriptome. Even if experiment-independent ML methods were trained using cell type-dependent or condition-specific training data, they would only discover modifications in those specifically pre-trained conditions. In contrast, experiment-based ML methods can detect changes in modifications between conditions and can be used to discover new patterns and functions for RNA modifications. However, since RNA modification detection in such approaches is performed on a specific dataset, the technical and biological variations in the experimental measurements must be considered. ML methods that can work on any sample and are robust to the variability of the experimental techniques will provide new opportunities to uncover the role of RNA modifications across cell types, tissues and other conditions.

### Scarcity and biases in training data

A limiting factor for training and testing ML methods to detect RNA modifications is the scarcity and variable quality of the available experimental data. For m6A, there are datasets with single-nucleotide resolution from multiple cell types and orthogonally validated sites using several experimental techniques. This has facilitated the development of ML algorithms to detect m6A sites at single-nucleotide resolution. In contrast, other RNA modifications have fewer or no datasets available, and often not at single-nucleotide resolution, which prevents the effective development of ML methods. Fewer experimentally validated sites also result in higher uncertainty about the expected accuracy of the algorithms. To understand the full extent of the epitranscriptome and create more reliable and comprehensive ML models, extensive data for all RNA modifications need to be generated. Furthermore, limited by the experimental techniques, modified sites with low stoichiometry or located in lowly expressed transcripts are still hard to detect. This also increases the difficulty of identifying the appropriate negative examples. For DRS-based ML tools, an alternative to biological sources for obtaining data to train models are IVTs. With IVTs, molecules can be made without modifications or substituting unmodified bases for their modified counterparts. DRS of these molecules yields a complete ground truth of signals for modified and unmodified nucleotides in the same sequence contexts, which can be used for training and testing new methods. As with any training strategy, biases contained in the dataset, and artifacts from experimental procedures and other sources will be passed on to the model. An alternative way to evaluate the performance of such models is to use data obtained using orthogonal techniques, but these present also their own biases and limitations. The coordinated efforts between computational and experimental researchers will therefore continue to be crucial to generate appropriate training and testing datasets.

### Opportunities and open questions in DRS

Well-studied RNAs, such as ribosomal and transfer RNAs, are known to harbor multiple modifications, and it is likely that this is common in mRNA too. DRS provides for the first time the theoretical possibility of measuring all RNA modifications present in each mRNA copy. However, it is not yet known how different nearby modifications in the same molecule affect the nanopore signal current. Specific training strategies incorporating combinations of modifications will be necessary to train and test model performance in realistic scenarios. These training and testing configurations are challenging to generate, and so far, only combinations of IVTs and cell line datasets have been used. Other challenges include the 3′ end bias and frequent 5′ end truncation in DRS. This results in a significant under-sampling of modifications in the 5′ untranslated regions, which are critical to understanding translation regulation. Another open question is whether there is a limitation in the type of modification and the resolution of the modifications that DRS can detect. Several current methods failed to separate m1A and m6A, two chemical isomers, based on their nanopore signals [24]. This may indicate that isomeric modifications and in general, modifications of similar physiochemical properties, may lead to very similar signal perturbations. Additional features besides signal values may be thus required to be able to distinguish these modifications using ML.

### Improvements in ML methods for RNA modification detection

The last few years have made patent that more computing power, larger training data and more complex architectures with more variables most likely increase the accuracy of DL models. Case studies in the computer vision [82] and natural language processing [83] fields have shown that an exponential increase in data and DL model sizes (i.e. the number of parameters) is needed to accomplish linear improvement, making it harder and harder to improve performance only focusing on these aspects of the ML task. On the other hand, the right use of biological information and model priors can increase the robustness and performance of ML models, possibly eliminating reliance on large training datasets. For example, DRS-based algorithms that detect RNA modifications do not yet explicitly consider information related to the relative transcript position, secondary structure or evolutionary conservation of the candidate nucleotide modification, which could inform the detection for many RNA modifications. As knowledge about RNA biology and RNA modifications is rapidly increasing, the correct encoding of this knowledge in ML models could improve their detection accuracy.

## CONCLUSIONS

ML methods play a central role in detecting RNA modifications. In this review, we outlined two main approaches used to detect RNA modifications with ML, experiment-independent and experiment-based methods. Their applicability mainly depends on the research question at hand. Experiment-independent methods are useful to investigate RNA modification sites in a similar condition as the training data or to obtain a general characterization of the epitranscriptome. Moreover, these algorithms can be easily interrogated to understand the relevant features of the modified sites. On the other hand, experiment-based tools will be more suitable to identify RNA modifications in new conditions that differ from the training data or to discover functional and regulatory aspects of the RNA modifications, guided by the experimental features. Both together provide an exciting framework for new discoveries and innovation in the field of epitranscriptomics.

**Key Points**

- This review highlights how machine learning provides effective strategies to identify and characterize RNA modifications at the transcriptome-wide level.
- Machine learning strategies are broadly categorized into two types based on the input data used for training and prediction, experiment-independent and experiment-based, and the advantages and applicability of each approach are discussed.
- The main strategies to train, test and interpret machine learning methods for RNA modifications are described.
- Current challenges and open questions about RNA modification analysis are presented, and the opportunities of machine learning to address these questions are discussed.
